# Neutrophil-to-lymphocyte ratio is a prognostic factor reflecting immune condition of tumor microenvironment in squamous cell lung cancer

**DOI:** 10.1038/s41598-023-50378-9

**Published:** 2024-01-03

**Authors:** Kana Ohashi, Yukari Nishito, Hironori Fukuda, Ryoichi Sadahiro, Yukihiro Yoshida, Shun-ichi Watanabe, Noriko Motoi, Yukiko Sonobe, Hideaki Mizuno, Hiroyuki Tsunoda, Koichiro Tatsumi, Takuji Suzuki, Atsushi Ochiai, Kazunori Aoki

**Affiliations:** 1 Department of Immune Medicine, National Cancer Center Research Institute, Tsukiji 5-1-1, Chuo-ku, Tokyo, 104-0045 Japan; 2 Kamakura Research Laboratories, Chugai Pharmaceutical Co., Ltd, 200 Kajiwara, Kamakura, Kanagawa 247-8530 Japan; 3 Department of Thoracic Surgery, National Cancer Center Hospital, Tsukiji 5-1-1, Chuo-ku, Tokyo, 104-0045 Japan; 4 Department of Diagnostic Pathology, National Cancer Center Hospital, Tsukiji 5-1-1, Chuo-ku, Tokyo, 104-0045 Japan; 5 Department of Respirology, Graduate School of Medicine, Chiba University, 1-8-1 Inohana, Chuo-ku, Chiba-shi, Chiba, 260-8677 Japan; 6 Exploratory Oncology Research and Clinical Trial Center, National Cancer Center, Kashiwanoha 6-5-1, Kashiwa, Chiba 277-8577 Japan

**Keywords:** Oncology, Cancer, Cancer, Cancer microenvironment, Lung cancer

## Abstract

Inflammatory factors in the peripheral blood, such as the C-reactive protein level and neutrophil-to-lymphocyte ratio (NLR), are prognostic markers in multiple types of cancer, including non-small cell lung cancer (NSCLC). However, the association between inflammatory factors and prognosis based on histological types has not been adequately reported. In addition, the relationship between these factors and the immune condition of the tumor microenvironment (TME) is unclear. In this single center, retrospective study, we first investigated the relationship between preoperative inflammatory markers and clinical outcomes in 176 patients with NSCLC who underwent surgery. Lung adenocarcinoma (LUAD) showed no significant prognostic marker, whereas for lung squamous cell carcinoma (LUSC), a multivariate analysis showed that a high NLR was significantly associated with postoperative recurrence. In LUSC patients, the median time of postoperative recurrence-free survival in patients with a low NLR was longer than that in patients with a high NLR. We then compared the tumor-infiltrating lymphocyte (TIL) profile with inflammatory markers in peripheral blood and found that the NLR was negatively correlated with the frequencies of T cells and B cells in LUSC tissues. Thus, the NLR is a useful predictive biomarker for postoperative recurrence and may reflect the immune condition of the TME in LUSC.

## Introduction

Lung cancer is histologically classified into small cell lung cancer (SCLC) and non-small cell lung cancer (NSCLC). SCLC accounts for approximately 15% of all lung cancers and is characterized by its neuroendocrine function. In contrast, NSCLC accounts for approximately 85% of lung cancers and includes multiple histological types, such as adenocarcinoma (LUAD), squamous cell carcinoma (LUSC), and large cell carcinoma. Among NSCLCs, LUAD and LUSC are the major histological subgroups and they have different genetic drivers, control networks, and prognostic profiles. In addition, clinical trials for NSCLC have shown different responses to chemotherapy, kinase mutation-targeted drugs, and immune checkpoint inhibitors in LUAD and LUSC^1,2^. Therefore, LUAD and LUSC are considered different diseases at the molecular, pathological, and clinical levels.

In multiple tumors, including lung cancer, inflammatory markers such as C-reactive protein (CRP), neutrophil-to-lymphocyte ratio (NLR), lymphocyte-to-monocyte ratio (LMR), and platelet-to-lymphocyte ratio (PLR) in peripheral blood have been reported to be significantly correlated with patient prognosis^3–8^. In SCLC and NSCLC patients, several reports examined the relationship between peripheral blood markers and prognosis^3,4,7^. However, to date, NSCLC has often been studied as a mixture of LUAD and LUSC to explore prognostic biomarkers, and few reports have compared the findings for many cases by dividing them into LUAD and LUSC.

The tumor-infiltrating lymphocyte (TIL) profile in the tumor microenvironment (TME) is associated with the prognosis of patients with NSCLC^9^. However it is not clear whether peripheral blood markers reflect TIL profiles in NSCLC tissues^8,11–18^. In this study, we first examined the association between inflammatory markers in peripheral blood and the prognosis in LUAD and LUSC. We then investigated the relationship between indicators of inflammation, which are prognostic predictors in peripheral blood, and the TIL profile, which reflects the immune condition of the TME.

## Results

### Clinicopathological characteristics of NSCLC patients

The baseline clinicopathological characteristics of the 176 NSCLC patients are summarized in Table 1. The median patient age was 70 years (range, 28–88 years), and 115 (65.3%) patients were male. Meanwhile, 137 (77.8%) patients had a history of smoking. According to the eighth edition of the TNM staging system, 66 (37.5%), 51 (29.0%), and 59 (33.5%) patients were categorized into stages I, II, and III, respectively. Of these patients, 101 (57.4%) had adenocarcinoma, 60 (34.1%) had squamous cell carcinoma, and 15 (8.5%) had other histological types. No patients received neoadjuvant therapy. 40 (22.7%) patients received postoperative chemotherapy, 2 (1.1%) patients received postoperative radiation therapy, and 1(0.6%) patient received postoperative chemoradiation. 56 (31.8%) patients showed recurrence while 120 (68.2%) patients did not. The median RFS was 24 months (range, 1–56 months). A total of 159 (90.3%) patients were alive and 17 (9.7%) patients died. The median OS was 28 months (range, 1 month; max, 56 months). On preoperative blood tests, the median CRP level and NLR, LMR, and PLR values for the study population were 0.13 (range, 0.01–9.22), 2.68 (0.67–48.66), 4.37 (1.48–9.25), and 154.2 (56.16–491.5), respectively.

**Table 1:** Clinicopathological characteristics of 176 patients with non-small cell lung cancer (NSCLC).

Characteristics	n (%)
Age	
Median (range)	70 (28–88)
< 70 years	82 (46.6)
≥ 70 years	94 (53.4)
Sex	
Male	115 (65.3)
Female	61 (34.7)
Smoking status	
Never	39 (22.2)
Former/Current	137 (77.8)
Pathological stage	
I	66 (37.5)
II	51 (29.0)
III	59 (33.5)
Histology	
Adenocarcinoma	101 (57.4)
Squamous cell carcinoma	60 (34.1)
Others	15 (8.5)
Adjuvant therapy	
Non	133 (75.6)
Adjuvant chemotherapy	40 (22.7)
Adjuvant radiation	2 (1.1)
Adjuvant chemotherapy + radiation	1 (0.6)
Recurrence	
Yes	56 (31.8)
No	120 (68.2)
Survey	
Alive	159 (90.3)
Died	17 (9.7)
CRP mg/dl (range)	0.13 (0.01–9.22)
NLR (range)	2.68 (0.67–48.66)
LMR (range)	4.37 (1.48–9.25)
PLR (range)	154.2 (56.16–491.5)

We then compared the clinicopathological characteristics between patients with LUAD (n = 101) and LUSC (n = 60). LUSC patients were older (*p* = 0.023), predominantly male (*p* < 0.001), and had a higher smoking status (p < 0.001), higher CRP levels (*p* < 0.001), and higher mortality rates (*p* < 0.001) than LUAD patients (Table 2). The LMR was higher (*p* = 0.001) in patients with LUAD than in those with LUSC.

**Table 2:** Comparison of clinicopathological characteristics between 101 patients with lung adenocarcinoma (LUAD) and 60 patients with lung squamous cell carcinoma (LUSC).

Characteristics	LUAD	LUSC	*P*-value
	n (%)	n (%)	
Age			
Median (range)	69 (28–88)	72 (50–88)	0.023
< 70 years	53 (52.5)	21 (35)	0.035
≥ 70 years	48 (47.5)	39 (65)	
Sex			
Male	54 (53.5)	51 (85)	< 0.001
Female	47 (46.5)	9 (15)	
Smoking status			
Never	35 (34.7)	1 (1.7)	< 0.001
Former/Current	66 (65.3)	59 (98.3)	
Pathological stage			
I	44 (43.6)	16 (26.7)	0.66
II	26 (24.8)	23 (38.3)	
III	32 (31.7)	21 (35)	
Adjuvant therapy			
Non	73 (72.3)	49 (81.6)	0.181
Adjuvant chemotherapy	27 (26.7)	9 (15)	(No vs. Yes)
Adjuvant radiation	1 (1.0)	1 (1.7)	
Adjuvant chemotherapy + radiation	0 (0)	1 (1.7)	
Recurrence			
Yes	29 (28.7)	18 (30)	0.86
No	72 (71.3)	42 (70)	
Survey			
Alive	99 (98)	47 (78.3)	< 0.001
Died	2 (2)	13 (21.7)	
CRP mg/dl (range)	0.08 (0.01–8.13)	0.3 (0.03–9.22)	< 0.001
NLR (range)	2.508 (0.67–48.66)	3.29 (1.21–18.3)	0.912
LMR (range)	4.708 (1.48–9.15)	3.25 (1.61–9.25)	0.001
PLR (range)	152.57 (69.12–491.52)	165.0 (56.16–415.85)	0.604

### Analysis of prognostic value for patient outcome

Univariate analysis of clinicopathological factors, inflammatory markers, and peripheral blood cell types showed that pathological stage, PLR, WBC and platelet counts, and neutrophil (%), lymphocyte (%), and monocyte (%) levels were correlated with RFS in NSCLC patients (Table 3). In patients with LUAD, only the pathological stage was correlated with RFS. In patients with LUSC, pathological stage, NLR, WBC count, neutrophil count, and neutrophil (%), lymphocyte (%), monocyte (%), and basophil levels (%) were correlated with RFS. Smoking status in patients with LUSC was indicated as not available (NA) because the number of nonsmokers was too small to be suitable for statistical analysis. Similarly, in Tables 4 and 5, the values for which the number of events was small and could not be analyzed are also indicated as NA.

**Table 3:** Univariate analysis of clinical characteristics on recurrent free survival (RFS).

Characteristics	NSCLC		LUAD		LUSC	
	(n = 176)		(n = 101)		(n = 60)	
	HR (95%CI)	q-value (*P*-value)	HR (95%CI)	q-value (*P*-value)	HR (95%CI)	q-value (*P*-value)
Age	0.983 (0.961–1.005)	0.295 (0.133)	0.988 (0.959–1.018)	0.675 (0.439)	0.964 (0.919–1.011)	0.254 (0.134)
Sex (Female vs. Male)	1.073 (0.617–1.885)	0.893 (0.804)	1.024 (0.492–2.13)	0.949 (0.949)	1.698 (0.389–7.411)	0.507 (0.481)
Smoking status (No vs. Yes)	0.738 (0.413–1.318)	0.469 (0.305	0.528 (0.255–1.093)	0.34 (0.085)	NA	–
Pathological stage	2.626 (1.821–3.758)	< 0.001 (< 0.001)	3.029 (1.816–5.053)	< 0.001 (< 0.001)	4.381 (1.889–10.16)	< 0.001 (< 0.001)
Adjuvant therapy (No vs. Yes)	1.529 (0.872–2.68)	0.276 (0.138)	1.725 (0.814–3.653)	0.387 (0.155)	0.855 (0.247–2.962)	0.805 (0.805)
CRP	1.159 (1.025–1.311)	0.233 (0.018)	1.171 (0.929–1.477)	0.404 (0.182)	1.111 (0.904–1.366)	0.465 (0.318)
NLR	1.015 (0.970–1.063)	0.682 (0.512)	0.986 (0.903–1.077)	0.878 (0.754)	1.445 (1.176–1.776)	< 0.001 (< 0.001)
LMR	0.878 (0.738–1.045)	0.262 (0.144)	0.931 (0.741–1.169)	0.716 (0.537)	0.791 (0.549–1.139)	0.357 (0.207)
PLR	1.005 (1.002–1.007)	0.007 (0.001)	1.004 (0.999–1.008)	0.295 (0.059)	1.005 (0.999–1.01)	0.139 (0.066)
WBC	1.139 (1.036–1.252)	0.002 (0.007)	1.029 (0.863–1.226)	0.883 (0.751)	1.262 (1.084–1.468)	0.014 (0.003)
RBC	0.997 (0.991–1.003)	0.437 (0.306)	0.999 (0.99–1.007)	0.834 (0.792)	0.996 (0.988–1.005)	0.510 (0.403)
Platelet	1.045 (1.012–1.078)	0.002 (0.007)	1.036 (0.975–1.1)	0.458 (0.252)	1.029 (0.982–1.078)	0.372 (0.235)
Neutrophil	1.003 (0.965–1.043)	0.907 (0.862)	0.973 (0.889–1.065)	0.689 (0.551)	1.332 (1.133–1.565)	< 0.001 (< 0.001)
Lymphocyte	0.723 (0.451–1.161)	0.045 (0.180)	0.658 (0.326–1.328)	0.486 (0.243)	0.713 (0.297–1.71)	0.502 (0.449)
Monocyte	1.652 (0.271–10.06)	0.732 (0.586)	0.237 (0.009–6.172)	0.643 (0.386)	4.053 (0.213–77.16)	0.477 (0.352)
Neutrophil (%)	1.056 (1.023–1.09)	< 0.001 (< 0.001)	1.034 (0.993–1.076)	0.297 (0.104)	1.088 (1.025 -1.156)	0.023 (0.006)
Lymphocyte (%)	0.942 (0.909–0.977)	0.007 (0.001)	0.961 (0.919–1.006)	0.290 (0.087)	0.919 (0.858–0.985)	0.046 (0.017)
Monocyte (%)	0.740 (0.599–0.913)	0.02 (0.005)	0.754 (0.566–1.005)	0.540 (0.054)	0.635 (0.419–0.962)	0.076 (0.032)
Eosinophil (%)	1.010 (0.871–1.172)	0.907 (0.892)	1.070 (0.872–1.314)	0.738 (0.517)	0.890 (0.673–1.176)	0.490 (0.413)
Basophil (%)	0.807 (0.296–2.202)	0.795 (0.676)	0.754 (0.566–1.005)	0.540 (0.054)	0.064 (0.007–0.566)	0.041 (0.013)

**Table 4:** Univariate analysis of clinical characteristics on overall survival (OS).

Characteristics	NSCLC (n = 176)		LUAD (n = 101)		LUSC (n = 60)	
	HR (95%CI)	q-value (*P*-value)	HR (95%CI)	q-value (*P*-value)	HR (95%CI)	q-value (*P*-value)
Age	1.018 (0.972–1.065)	0.679 (0.453)	1.161 (0.947 -1.425)	1.283 (0.151)	0.965 (0.914–1.02)	0.440 (0.207)
Sex (Female vs. Male)	8.755 (1.161–66.02)	0.126 (0.035)	NA	–	NA	–
Smoking status (No vs. Yes)	NA	–	NA	–	NA	–
Pathological stage	2.306 (1.202–4.422)	0.108 (0.012)	2.503 (0.384–16.31	1.915 (0.338)	4.729 (1.667–13.41)	0.051 (0.003)
Adjuvant therapy (No vs. Yes)	NA	–	NA	–	NA	–
CRP	1.262 (1.058–1.505)	0.180 (0.010)	0.784 (0.072–8.574)	1.022 (0.842)	1.151 (0.924–1.435)	0.397 (0.210)
NLR	1.006 (0.913–1.11)	0.897 (0.897)	0.621 (0.144–2.677)	1.481 (0.523)	1.137 (0.951–1.36)	0.453 (0.160)
LMR	0.849 (0.617–1.169)	0.517 (0.316)	1.186 (0.530–2.653)	0.960 (0.678)	0.892 (0.598–1.33)	0.610 (0.574)
PLR	1.001 (0.996–1.007)	0.769 (0.641)	0.988 (0.958–1.019)	1.486 (0.437)	1.005 (0.999–1.011)	0.497 (0.117)
WBC	1.192 (1.032–1.376)	0.102 (0.017)	0.928 (0.415–2.072)	0.969 (0.855)	1.070 (0.879–1.303)	0.609 (0.502)
RBC	0.991 (0.980–1.001)	0.200 (0.078)	1.005 (0.974–1.037)	0.977 (0.747)	0.995 (0.984–1.005)	0.445 (0.314)
Platelet	1.046 (0.987–1.108)	0.262 (0.131)	0.886 (0.662–1.185)	1.759 (0.414)	1.019 (0.959–1.083)	0.618 (0.545)
Neutrophil	1.010 (0.950–1.073)	0.804 (0.759)	0.771 (0.247–2.41)	1.012 (0.655)	1.106 (0.914–1.339)	0.462 (0.299)
Lymphocyte	1.271 (0.5940–2.72)	0.689 (0.536)	1.17 (0.111–12.32)	0.896 (0.896)	0.627 (0.208–1.886)	0.531 (0.406)
Monocyte	12.71 (0.865–186.6)	0.192 (0.064)	0.033 (< 0.001–19950)	1.045 (0.615)	1.428 (0.035–57.76)	0.850 (0.850)
Neutrophil (%)	1.046 (0.989–1.107)	0.263 (0.117)	0.959 (0.836–1.1)	1.340 (0.552)	1.063 (0.997–1.133)	0.357 (0.063)
Lymphocyte (%)	0.954 (0.896–1.017)	0.266 (0.148)	1.015 (0.862–1.195)	0.913 (0.859)	0.948 (0.879–1.023)	0.405 (0.167)
Monocyte (%)	0.886 (0.616–1.274)	0.710 (0.513)	0.754 (0.251–2.264)	1.160 (0.614)	0.755 (0.482–1.182)	0.372 (0.219)
Eosinophil (%)	0.938 (0.707–1.243)	0.737 (0.655)	2.362 (1.05–5.313)	0.646 (0.038)	0.747 (0.509–1.096)	0.462 (0.136)
Basophil (%)	0.106 (0.014–0.821)	0.144 (0.032)	3.236 (0.054–194.5)	1.220 (0.574)	0.026 (0.002–0.328)	0.042 (0.005)

**Table 5:** Multivariate analysis of clinical characteristics on RFS and OS.

(A) Multivariate analysis of clinical characteristics on RFS						
Characteristics	NSCLC (n = 176)		LUAD (n = 101)		LUSC (n = 60)	
	HR (95%CI)	q-value (*P*-value)	HR (95%CI)	q-value (*P*-value)	HR (95%CI)	q-value (*P*-value)
Age	0.999 (0.972–1.027)	0.957 (0.957)	1.012 (0.971–1.055)	1.040 (0.578)	0.952 (0.888–1.021)	0.272 (0.170)
Sex (Female vs. Male)	1.148 (0.554–2.379)	0.800 (0.711)	1.237 (0.476–3.210)	0.852 (0.663)	1.958 (0.373–10.29)	0.488 (0.427)
Smoking status (No vs. Yes)	0.749 (0.355–1.581)	0.806 (0.448)	0.535 (0.217–1.320)	0.394 (0.175	NA	–
Pathological stage	2.645 (1.786–3.919	< 0.001 (< 0.001)	3.377 (1.897–6.012)	< 0.001 (< 0.001)	3.694 (1.512–9.023)	0.032 (0.004)
Adjuvant therapy (No vs. Yes)	0.749 (0.380–1.477)	0.909 (0.404)	0.780 (0.271–2.250)	0.769 (0.646)	0.152 (0.021–1.091)	0.122 (0.061)
CRP	1.043 (0.901–1.206)	0.735 (0.572)	1.057 (0.810–1.380)	0.769 (0.684)	0.878 (0.584- 1.318)	0.529 (0.529)
NLR	1.024 (0.951–1.101)	0.799 (0.533)	0.989 (0.813–1.204)	0.916 (0.916)	2.262 (1.230–4.160)	0.036 (0.009)
LMR	1.133 (0.914–1.406)	0.762 (0.254)	1.274 (0.924–1.755)	0.417 (0.139)	1.300 (0.762–2.217)	0.447 (0.335)
PLR	1.004 (1.001–1.00)	0.085 (0.019)	1.006 (1.000–1.011)	0.222 (0.049)	0.989 (0.979–0.999)	0.067 (0.025)

(B) Multivariate analysis of clinical characteristics on OS						
Characteristics	LUSC (n = 60)					
	HR (95%CI)	q-value (*P*-value)				
Age	0.920 (0.833–1.016)	0.150 (0.100)				
Sex (Female vs. Male)	NA	–				
Smoking status (No vs. Yes)	NA	–				
Pathological stage	4.925 (1.304–18.60)	0.114 (0.019)				
Adjuvant therapy (No vs. Yes)	NA	–				
CRP	1.311 (0.980–1.755)	0.204 (0.068)				
NLR	1.423 (0.403–5.027)	0.701 (0.584)				
LMR	1.944 (0.920–4.108)	0.162 (0.081)				
PLR	0.996 (0.980–1.012)	0.624 (0.624)				

In univariate analysis of the prognostic value for OS, we found that there was no factor correlated with OS in NSCLC and LUAD, and only the basophil (%) were correlated with OS in patients with LUSC.

In the multivariate analysis, worse RFS was associated with a high pathological stage (hazard ratio [HR], 3.377; 95% CI 1.897–6.012; q < 0.001; *p* < 0.001) in LUAD patients (Table 5A). In LUSC patients, RFS was significantly worse in patients with high pathological stage (HR, 3.694; 95% CI, 1.512–9.023; q = 0.032; *p* = 0.004) and NLR (HR, 2.262; 95% CI 1.23–4.16; q = 0.036, *p* = 0.009) (Table 5A).

In LUAD patients, multivariate analysis for OS was not performed because of the small number of patients who died, and no factor was correlated with OS in patients with LUSC (Table 5B).

**Figure 1:**
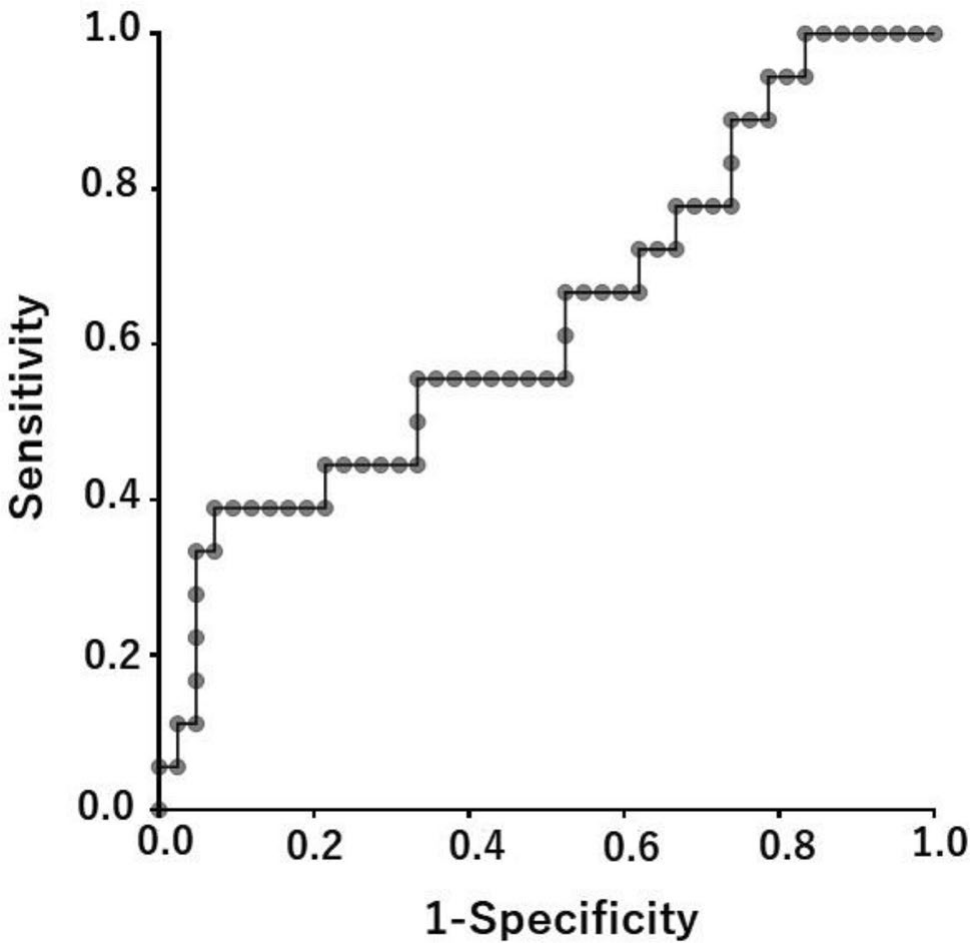
ROC curve of NLR in postoperative recurrence in patients with squamous cell carcinoma of the lung. Cut-off value: 4.787, sensitivity: 38.9%, specificity: 92.9%, AUC: 0.634. ROC, receiver operating characteristic; AUC, area under curve.

**Figure 2:**
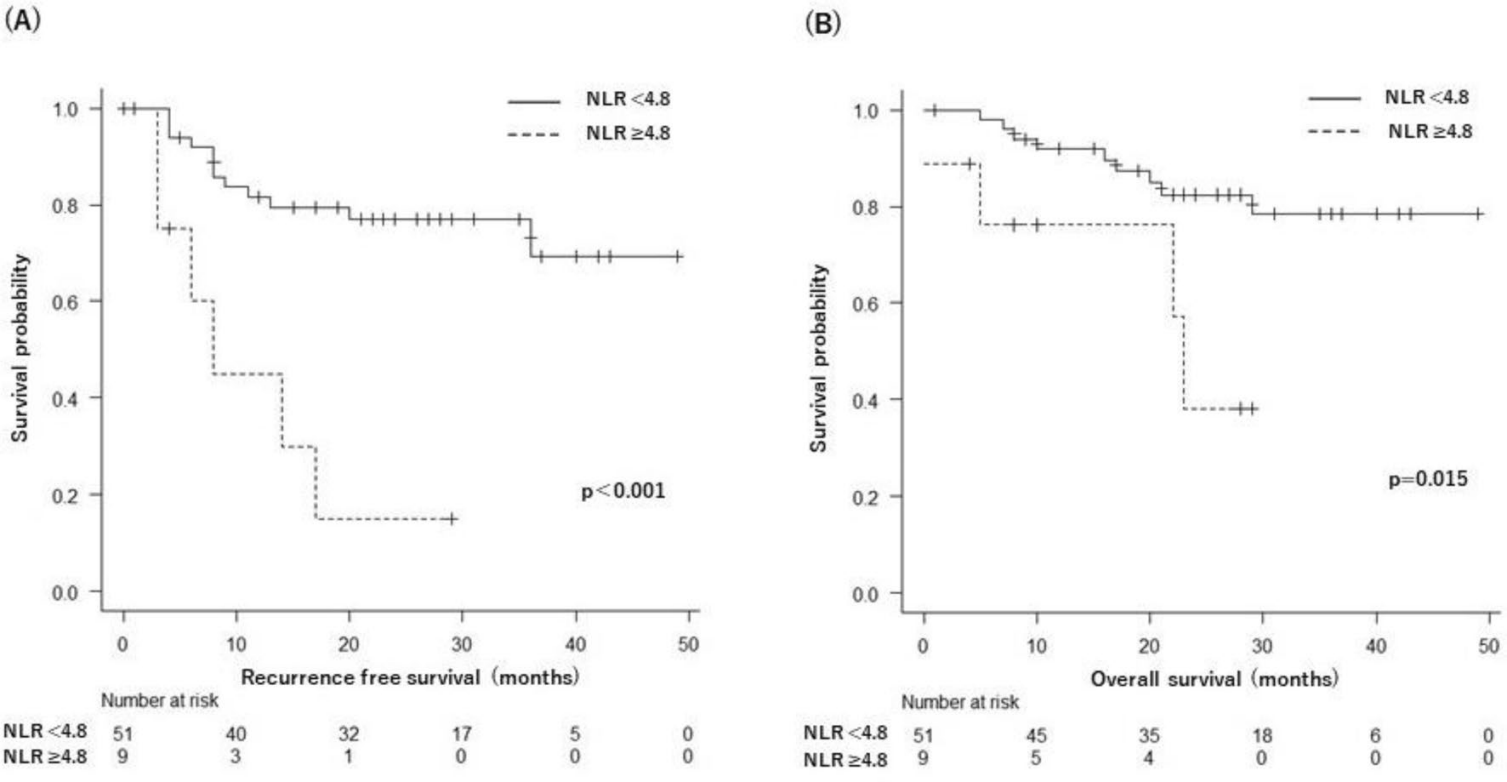
Kaplan–Meier survival curves according to preoperative neutrophil-to-lymphocyte ratio in patients with LUSC. (**A**) Recurrence-free survival. (**B**) Overall survival.

We then examined the cutoff NLR value to assess its clinical performance. The ROC curve showed that the best NLR cut-off value was 4.787 (Fig. 1). For this cutoff value, the sensitivity was 38.9%, specificity was 92.9%, and the area under the curve (AUC) was 0.634. The cutoff value for NLR was set at 4.8 as an approximation, and 51 (85%) LUSC patients were categorized into the high NLR group (NLR ≥ 4.8), while the remaining 9 (15%) LUSC patients were stratified into the low NLR group (NLR < 4.8). LUSC patients with a low NLR had a longer RFS than those with a high NLR (NA vs. 8 months, *p* < 0.001) (Fig. 2A). Similarly, patients with LUSC with a low NLR had better OS than those with a high NLR (NA vs. 23 months, *p* = 0.015) (Fig. 2B).

### Association between NLR and the TIL profile in lung cancer

**Figure 3:**
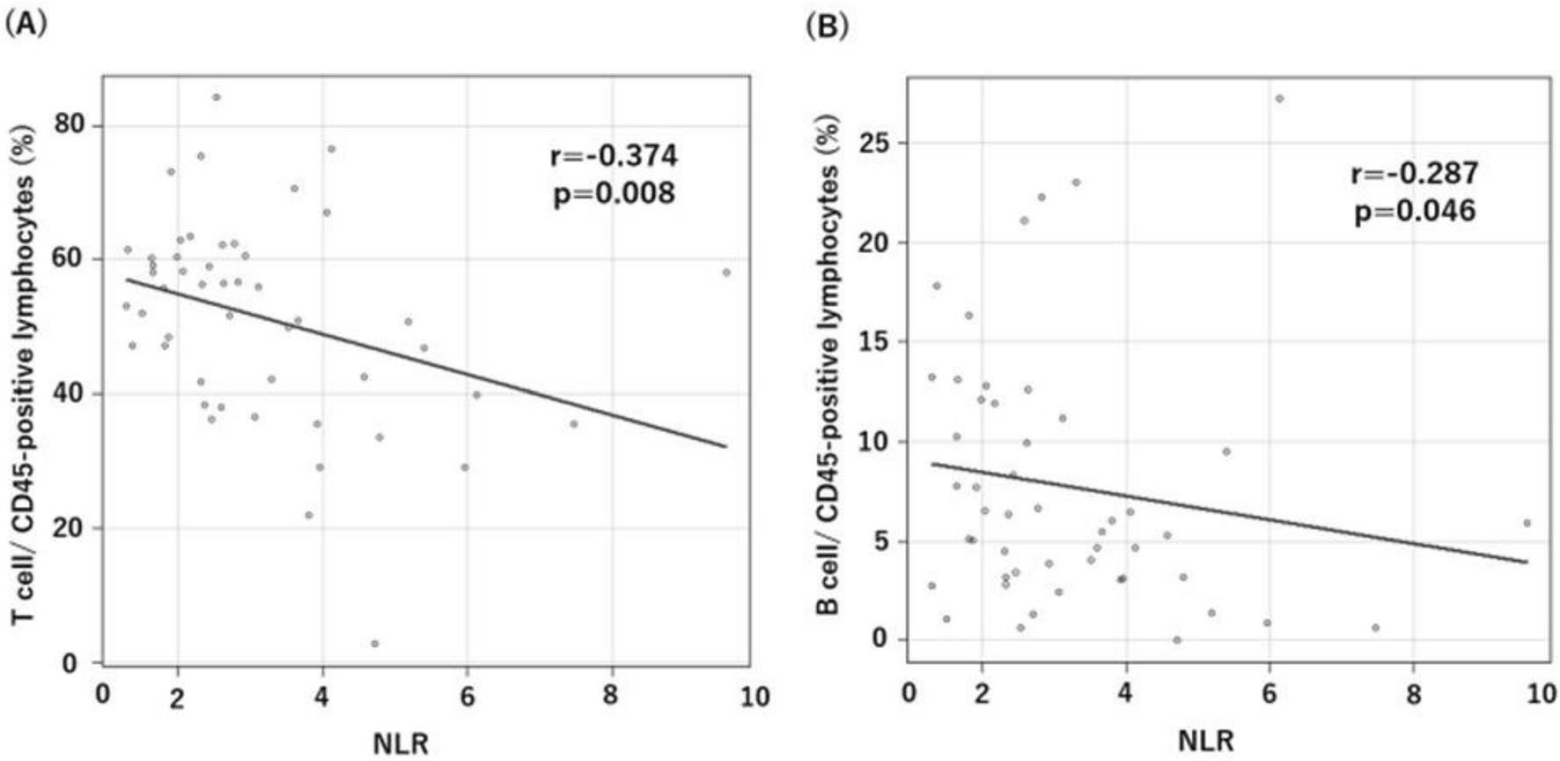
Correlation between the NLR in preoperative blood tests and the TIL status in the TEM in patients with LUSC. (**A**) Correlation of the NLR value with T cells/CD45-positive lymphocytes in TILs. (**B**) Correlation of the NLR value with B cells/CD45-positive lymphocytes in TILs.

To examine whether NLR reflects the immune condition of the TME, we evaluated the correlations between NLR and the TIL profile. To analyze the TIL profile, we employed a flow cytometry (FCM) panel of 26 markers to identify 13 unique immune cell types and functional subpopulations from 140 NSCLC samples (82 LUAD samples, 49 LUSC samples, and 9 other histological subtype samples). Flow cytometry showed that NLR was negatively correlated with the frequencies of T cells/CD45^+^ cells (r = − 0.374, *p* = 0.008) and B cells/CD45^+^ cells (r = − 0.287, p = 0.046) in the tumor tissues of LUSC patients (Fig. 3). NLR showed a negative correlation with the frequency of CD8^+^ T cells/CD45^+^ cells (r = − 0.265, *p* = 0.066) and a positive correlation propensity with macrophages/CD45^+^ cells (r = 0.259, *p* = 0.072) (Table 6). No obvious correlation was found between other TIL immune cells and NLR in patients with LUSC (Table 6). NLR showed a positive correlation with non-Tregs (Fr. III)/CD45^+^ cells (r = 0.364, *p* < 0.001) in patients with LUAD^10^.

**Table 6:** Association between NLR and TIL profile in lung cancer.

Immune cell	LUAD (n = 82)	LUSC (n = 49)
T cells/CD45^+^ cells	r = 0.088	r = − 0.374
	*p* = 0.429	*p* = 0.008
CD8^+^ T cells/CD45^+^ cells	r = − 0.104	r = − 0.265
	*p* = 0.35	*p* = 0.066
CD4^+^ T cells/CD45^+^ cells	r = − 0.166	r = − 0.238
	*p* = 0.137	*p* = 0.099
Naïve Tregs (Fr. I)/CD45^+^ cells	r = 0.089	r = 0.007
	*p* = 0.428	*p* = 0.959
Effector Tregs (Fr. II)/CD45^+^ cells	r = 0.171	r = 0.009
	*p* = 0.124	*p* = 0.948
Non-Tregs (Fr. III)/CD45^+^ cells	r = 0.364	r = − 0.11
	*p* < 0.001	*p* = 0.449
NKT cells/CD45^+^ cells	r = − 0.139	r = − 0.022
	*p* = 0.213	*p* = 0.881
B cells/CD45^+^ cells	r = 0.003	r = − 0.287
	*p* = 0.98	*p* = 0.046
NK cells/CD45^+^ cells	r = − 0.07	r = − 0.106
	*p* = 0.532	*p* = 0.467
Conventional DC/CD45^+^ cells	r = 0.064	r = 0.189
	*p* = 0.565	*p* = 0.192
Plasma cell DC/CD45^+^ cells	r = − 0.024	r = − 0.029
	*p* = 0.832	*p* = 0.843
Macrophages/CD45^+^ cells	r = 0.007	r = 0.259
	*p* = 0.953	*p* = 0.072
Monocytic MDSC/CD45^+^ cells	r = − 0.044	r = 0.125
	*p* = 0.694	*p* = 0.391

## Discussion

NLR has been reported to be associated with the systemic inflammatory status^11–16^, which increases the number of neutrophils and affects tumor growth and progression^17^. Lymphocytes are essential immune cells in both humoral and cellular antitumor immune responses, and low lymphocyte counts are associated with an immunosuppressive condition in patients with cancer^18–20^. Therefore, a high NLR has been considered to be a predictor of poor prognosis in many cancers, including NSCLC^3,8^. However, few studies have reported comparisons of prognostic factors in relation to the histological type of NSCLC. This is the first study to show that prognostic markers in the peripheral blood of patients showing postoperative recurrence differ by histologic type and to suggest that NLR, as a prognostic marker, reflects the tumor microenvironment in LUSC, which was clarified by comparing the findings for LUAD and LUSC^21^.

TILs are associated with the prognosis of multiple cancers. CD8^+^ T cells ultimately differentiate into cytotoxic T cells, which have cell-killing functions^22,23^. CD4^+^ T cells are required for nearly all functions in tumor immunity^24^. Recent studies have also identified a key role of B lymphocytes in immunotherapy, and their presence has been associated with an improved prognosis across different cancer types^25,26^. Since peripheral blood obtained using a minimally invasive technique is an ideal biomarker, identification of the peripheral blood factors that correlate with T and B cells in the TME will be of value. Several reports have shown a negative correlation between NLR and CD3^+^ cells in TILs in multiple cancers, including NSCLC^7,27^. Our study showed that the NLR is negatively correlated with T cells as well as B cells in TILs for LUSC but not for LUAD patients, explaining that NLR is a poor prognostic factor in LUSC patients.

TIL composition and activation status were associated with patient outcomes^28^. TILs were mainly evaluated by immunohistochemical analyses focusing on CD3^+^ cells and CD8^+^ T cells^7,29,30^ and morphological evaluation by HE staining^31,32^. However, TIL analysis using FCM has been described in only a few reports^33^. In this study, we examined multiple unique immune cell types and functional subpopulations by FCM, which allowed simultaneous comparison of multiple TIL immune cell types with NLR.

Although not analyzed in this study, tumor-associated neutrophils (TANs) were present in the TME. TANs occur in two states: antitumor (N1) and tumor growth-promoting (N2)^34^. Because of the positive correlation between TANs and peripheral blood neutrophil counts, a high NLR may be a poor prognostic factor, reflecting N2 neutrophils in the TME^35^.

For the treatment of lung cancer, in addition to anti-PD-1/PD-L1 antibody therapy, combined therapy with chemotherapy and immunotherapy has become the standard of care^36–41^. The development of predictive biomarkers for the efficacy of immune-checkpoint inhibitors (ICBs) in the peripheral blood is awaited. NLR has been reported to predict the efficacy of immune checkpoint therapy in NSCLC patients^42–44^. Since TIL compositions influence the efficacy of ICBs, this study may contribute to understanding the reason why NLR is a prognosis predictor for ICBs.

This study had several limitations: the sample size was not large, the observation period was not long, and the relationship with the efficacy of ICBs was not examined. More research is needed to confirm the usefulness of the NLR as a prognostic factor and ICB-effect predictor in a large cohort. To understand why prognostic factors in the peripheral blood differ between LUAD and LUSC, the differences in the TME between LUAD and LUSC and the relationship between peripheral blood factors and the TME require elucidation. Furthermore, there are also possible limitations regarding the statistical analysis. For the NLR cutoff, the ROC curve does not account for time factors. It may have been more desirable to perform the analysis using survival-ROC analysis^45^.

In conclusion, a high NLR was significantly associated with poor prognosis in LUSC but not in LUAD patients, reflecting the frequencies of T and B cells in the TME.

## Material and methods

### Patients

This retrospective study enrolled 176 patients with pathological stage I-III primary NSCLC who underwent surgery at the National Cancer Center Hospital (Tokyo, Japan) between October 2016 and June 2019. Patients with metastatic or pathological stage IV disease were excluded. This study protocol was approved by the National Cancer Center Ethics Committee (2016–124, dated: August 5th, 2016). All patients provided written informed consent before sampling. The study also abided by the principles of the Declaration of Helsinki.

Adjuvant therapies and neoadjuvant therapies were examined by multidisciplinary discussions for each individual patient based on pathologic findings, patient performance status, age, comorbidity and patient’s intension.

### Clinical follow-up

Recurrence-free survival (RFS) was defined as the interval between surgery and disease progression or death, whichever occurred first. Patients without any of these events were censored at the final follow-up, without documented progression. Overall survival (OS) was defined as the time from surgery to death from any cause. Patients without any of these events were censored at the final follow-up visit. The median follow-up period for all patients was 24 months (range, 1–56 months). The median follow-up period for the surviving patients was 28 months (range, 1–56 months).

### Blood inflammatory markers

Blood samples were collected the day before surgery. The NLR was defined as the number of neutrophils divided by the number of lymphocytes. LMR was defined as the lymphocyte count divided by the monocyte count, and PLR was defined as the platelet count divided by the lymphocyte count.

### Flow cytometry

Viable cells from tumor suspensions were counted and incubated with Fixable Viability Dye eFluor™ 506 (BioLegend, San Diego, CA, USA) for 30 min at 4 °C for dead cell staining, followed by FcR blocking using the FcR blocking reagent (Miltenyi Biotech) for 10 min at 4 °C. The cells were then stained with the fluorescently labeled antibodies listed in Table S1 at 4 °C for 30 min. Next, intracellular staining was performed with intracellular antibodies and a Foxp3/Transcription Factor Staining Buffer set (Thermo Fisher Scientific) according to the manufacturer's instructions.

After washing, the cells were analyzed using an LSR Symphony instrument (BD Biosciences, Franklin Lakes, NJ, USA). At least 50,000 live events were collected per sample (BD LSR II cytometer). Data were analyzed using the FlowJo software (BD Biosciences). The gating of each sample was based on plots of SSC-Height versus SSC-Width and FSC-Height versus FSC-Width to eliminate aggregates. FVD staining was used to identify and eliminate dead cells as evaluated using contour plots. A propidium iodide overlay was used to validate cell viability in the training set.

The 13 immune cells are defined as shown in Supplementary Table 2. The frequency of each immune cell in the TILs was calculated as the number of transformed cells divided by the number of CD45^+^ cells.

### Statistical analysis

All statistical analyses were conducted using R version 4.0.1 (R Foundation for Statistical Computing, Vienna, Austria). Statistical significance was set at *p* < 0.05. Cox proportional hazards regression models were used to evaluate the prognostic factors in univariate and multivariate analyses. The p value was adjusted by the False Discovery Rate with 5% significant level. Cutoff values were calculated using receiver operating characteristic (ROC) curves for factors considered significant prognostic markers. RFS and OS curves were analyzed using the Kaplan–Meier method, and statistical differences were determined using the log-rank test. Correlations between immune cells in TILs and inflammatory markers in peripheral blood were calculated using Spearman's rank correlation coefficient test.

## Supplementary Information

Supplementary Tables.

## Data Availability

The datasets used and/or analyzed during the current study available from the corresponding author on reasonable request.
