# Author Correction: Neutrophil-to-lymphocyte ratio is a prognostic factor reflecting immune condition of tumor microenvironment in squamous cell lung cancer

**DOI:** 10.1038/s41598-024-81463-2

**Published:** 2024-12-13

**Authors:** Kana Ohashi, Yukari Nishito, Hironori Fukuda, Ryoichi Sadahiro, Yukihiro Yoshida, Shun-ichi Watanabe, Noriko Motoi, Yukiko Sonobe, Hideaki Mizuno, Hiroyuki Tsunoda, Koichiro Tatsumi, Takuji Suzuki, Atsushi Ochiai, Kazunori Aoki

**Affiliations:** 1 Department of Immune Medicine, National Cancer Center Research Institute, Tsukiji 5-1-1, Chuo-ku, Tokyo, 104-0045 Japan; 2 Kamakura Research Laboratories, Chugai Pharmaceutical Co., Ltd, 200 Kajiwara, Kamakura, Kanagawa 247-8530 Japan; 3 Department of Thoracic Surgery, National Cancer Center Hospital, Tsukiji 5-1-1, Chuo-ku, Tokyo, 104-0045 Japan; 4 Department of Diagnostic Pathology, National Cancer Center Hospital, Tsukiji 5-1-1, Chuo-ku, Tokyo, 104-0045 Japan; 5 Department of Respirology, Graduate School of Medicine, Chiba University, 1-8-1 Inohana, Chuo-ku, Chiba-shi, Chiba, 260-8677 Japan; 6 Exploratory Oncology Research and Clinical Trial Center, National Cancer Center, Kashiwanoha 6-5-1, Kashiwa, Chiba 277-8577 Japan

Correction to: *Scientific Reports* 10.1038/s41598-023-50378-9, published online 03 January 2024

The original version of this Article contained an error in Reference 45, which was incorrectly given as:

Frencesco, J. R. *et al*. Neutrophil-to-lymphocyte ratio is a major prognostic factor in non-small cell lung carcinoma patients undergoing first line immunotherapy with pembrolizumab. *Cancer Diagn. Progn*. **3**, 44–52 (2023).

The correct reference is listed below:

Romano, F. J. et al. Neutrophil-to-lymphocyte ratio is a major prognostic factor in non-small cell lung carcinoma patients undergoing first line immunotherapy with pembrolizumab. *Cancer Diagn. Progn*. **3**(1), 44–52. 10.21873/cdp.10178 (2023).

The original Article has been corrected.

