# Supplementary material for: Neutrophil-to-lymphocyte ratio is a prognostic factor reflecting immune condition of tumor microenvironment in squamous cell lung cancer
Source: Sci Rep. 2024 Jan 3;14:429. doi: 10.1038/s41598-023-50378-9 (PMC10764784; doi:10.1038/s41598-023-50378-9)
Supplement: Supplementary file 1 — Supplementary Tables. [file 41598_2023_50378_MOESM1_ESM.pdf]

Supplementary Table 1. Antibodies for FACS

Target Antigen	Ab clone	Isotype	Label	manufacturer	Cat. No.
CD16	3G8	mIgG1k	BUV805	BD	624287
CD152	BNI3	mIgG2ak	BUV737	BD	624286
HLA-DR	G46-6	mIgG2ak	BUV661	BD	565073
CD86	2331	mIgG1k	BUV615	BD	624297
CD8	RPA-T8	mIgG1k	BUV563	BD	565695
CD19	SJ25C1	mIgG1k	BUV496	BD	564655
CD123	7G3	mIgG2ak	BV786	BD	564196
CD11b	ICRF44	mIgG1k	BV750	BD	624380
CD68	Y1/82A	mIgG2bk	BV711	BD	565594
CD56	NCAM16.2	mIgG2bk	BV650	BD	564057
CD326	9C4	mIgG2bk	BV605	Biolegend	324224
CD4	SK3	mIgG1k	BV570	BD	624298
L/D	-	-	BV510	invitrogen	L34965
CD11c	B-Ly6	mIgG1k	BB790	BD	624296
CD279	EH12.1	mIgG1k	BB700	BD	566460
CD163	GHI/61	mIgG1k	BB660	BD	624295
CD3	SK7	mIgG1k	BB630	BD	624294
CD274	MIH1	mIgG1k	PE-Cy7	BD	558017
CD14	TuK4	mIgG2a	PE-Cy5.5	invitrogen	MHCD1418
CD206	19.2/ 15-2	mIgG1k	PE-Cy5	BD/ Biolegend	551136/ 321108
CD33	WM53	mIgG1k	PE-CF594	BD	562492
FoxP3	206D	mIgG1k	PE	Biolegend	320108
CD45	2D1	mIgG1k	APC-H7	BD	560178

Supplementary Table 2. Defining immune cells in the TME

Immune cells	maker
T cell	$CD3^{+}CD56^{-}$
$CD8^{+}$ T cell	$CD3^{+}CD8^{+}$
$CD4^{+}$ T cell	$CD3^{+}CD4^{+}$
naïve Tregs (Fr. I)	$CD45RA^{+}$FOXP3 low
effector Tregs (Fr. II)	$CD45RA^{-}$FOXP3 high
non-Tregs (Fr. III)	$CD45RA^{-}$FOXP3 low
NKT cell	$CD3^{+}CD56^{+}$
B cell	$CD3^{-}CD19^{+}$
NK cell	$CD3^{-}CD56^{+}$
conventional DC	$HLA\text{-}DR^{+}CD11c^{+}$
plasma cell DC	$HLA\text{-}DR^{+}CD11c^{-}CD123^{+}$
macrophages	$CD68^{+}$SSC high
monocytic MDSC	HLA-DR low $CD14^{+}CD11b^{+}CD33^{+}$
